# Cerium oxide nanoparticles enhance rice productivity by modulating nitric oxide signaling, carbon metabolism, and potassium homeostasis under chromium-induced environmental stress

**DOI:** 10.3389/fpls.2026.1779968

**Published:** 2026-03-05

**Authors:** Haider Sultan, Mukerrem Atalay Oral, Gulsah Bengisu, Resat Esgici, Mohammad Faizan, Abdulrahman A. Alatar, Changli Zeng, Mohammad Faisal

**Affiliations:** 1 Hubei Engineering Research Center for Protection and Utilization of Special Biological Resources in the Hanjiang River Basin, College of Life sciences, Jianghan University, Wuhan, Hubei, China; 2 Kemer Faculty of Maritime Studies, Akdeniz University, Antalya, Türkiye; 3 Department of Field Crops, Faculty of Agriculture, Harran University, Sanliurfa, Türkiye; 4 Bismil Vocational High School, Dicle University, Diyarbakir, Türkiye; 5 Botany Section, School of Sciences, Maulana Azad National Urdu University, Hyderabad, India; 6 Department of Botany & Microbiology, College of Science, King Saud University, Riyadh, Saudi Arabia

**Keywords:** carbohydrate metabolism, environmental protection, nanotechnology, seed priming, sustainable agriculture

## Abstract

Chromium (Cr) contamination in agricultural soils represents a serious challenge to rice productivity and food security, underscoring the need for innovative approaches to improve Cr-tolerance and alleviate its detrimental effects on plant growth and development. Recently, nanotechnology has been increasingly used to improve the tolerance of plants exposed to metal stress. In view of the beneficial roles of nanoparticles in mitigating metal stress in plants, this study was conducted to assess the effectiveness of cerium dioxide nanoparticles (CeO_2_ NPs) in alleviating Cr-induced toxicity in rice plants. Chromium stress (100 µM) and CeO_2_ NPs (100 ppm) were applied at the seed stage by soaking the seeds in their respective solutions for 12 h prior to sowing. Chromium stress markedly increased the contents of thiobarbituric acid-reactive substances (TBARS) by 67%, hydrogen peroxide (H_2_O_2_) by 62%, superoxide anion (O_2_•^-^) by 58%, and methylglyoxal (MG) by 63% compared with control plants. In contrast, Cr stress significantly reduced the concentrations of starch (51%), sucrose synthase (49%), amylase (68%), chlorophyll (69%), and RuBisCO (67%). However, the application of CeO_2_ NPs markedly enhanced plant growth and biomass accumulation, alleviated oxidative stress, and stimulated antioxidant enzyme activities in rice plants subjected to Cr stress. Overall, these results demonstrate that the application of CeO_2_ effectively alleviates Cr-induced stress in rice plants and offers promising prospects for advancing NP-based phytoremediation strategies.

## 1 Introduction

Following the industrial revolution, there has been a significant escalation in global pollution, particularly heavy metal (HM) contamination, resulting from various anthropogenic activities such as extensive mining, smelting, the use of chemical fertilizers and explosives, electroplating, automobile combustion, dam construction, electricity generation, and unplanned drainage systems for irrigation (Arif et al., 2016; Balali-Mood et al., 2021; Chen et al., 2022; Bice et al., 2026). These activities not only contribute to HM pollution but also serve as primary drivers of climate change, intensifying both biotic and abiotic stresses in the environment (Chaudhry and Sidhu, 2021; Zandalinas et al., 2021; Faisal et al., 2025). Apart from natural processes like rock weathering and volcanic eruptions, anthropogenic activities have significantly elevated HM levels, posing a substantial threat to biodiversity, agricultural lands, and crop productivity (Alengebawy et al., 2021). HM stress in agricultural lands is particularly critical as it diminishes crop growth and productivity, consequently impacting farmers’ income (Chebout et al., 2023; Faizan et al., 2025). The uptake of non-essential elements by plants from the growth medium, primarily soil or water, through essential element pathways leads to toxicity, disrupting plant metabolism (Ali et al., 2023; Faizan et al., 2024).

Chromium (Cr) is one of the most toxic metals in environment which contaminates the ground water and soil due to its wide use in industry. Among the different valence states of chromium, hexavalent chromium (Cr^6+^) is predominantly used in plant stress studies due to its high stability, toxicity, and mobility. Upon cellular reduction, Cr^6+^ is converted to trivalent chromium (Cr^3+^), a process that generates reactive oxygen species responsible for cytotoxic, genotoxic, and physiological disturbances (Nigam et al., 2014). Plants primarily uptake Cr^6+^ through the plasma membrane via energy-dependent active transport mechanisms involving phosphate and sulfate transporters (Ali et al., 2023). Studies have reported the translocation of Cr^6+^ Cr from root to shoot is mediated by sulfur accumulators and by iron accumulators (Ali et al., 2023). Plants exposed to Cr stress manifest symptoms such as chlorosis, bronzing, and necrosis, leading to compromised growth and reduced production (Anjum et al., 2017). Decrease in the transpiration rate was reported in Lolium perenne leaves due to chromium toxicity. Chromium also disturbs mineral uptake and water imbalance in the cells (Gill et al., 2015). Cr toxicity, particularly its inhibitory effect on the enzyme δ-aminolevulinic acid dehydratase (ALAD) involved in chlorophyll biosynthesis, disrupts the utilization of δ -aminolevulinic acid. This impairment extends to the electron transport system in photosystem II and CO_2_ assimilation, resulting in a low photosynthetic rate. Additionally, it promotes stomatal closure, reduces the net photosynthetic rate, and lowers transpiration and respiration rates, ultimately leading to diminished biomass production (Shilin et al., 2022).

Reactive oxygen species (ROS) produced during chromium toxicity such as O_2_-, H_2_O_2_, and hydroxyl radicals (•OH) disrupt normal metabolic functions in plants. The increased ROS activity disrupts the cellular structures like protein, lipids and nucleic acids (Daud et al., 2021; Alam et al., 2025). Plants express antioxidant enzymes such as superoxide dismutase (SOD), peroxidase (POD), catalase (CAT) to detoxify ROS. However, the activity of these antioxidant enzymes decreases in Cr (Daud et al., 2021) and other HM-stressed plants. Increase in MDA concentration which is an indicator of oxidative damage caused by enhanced ROS activity was observed in *Triticum aestivum* exposed to higher Cr concentrations (Panda and Choudhury, 2005).

Nanotechnology represents an advanced and promising approach in agricultural science for enhancing crop productivity. The application of nanoparticles (NPs) in agriculture has been extensively documented, with several inorganic NPs shown to mitigate plant abiotic stress through the enhancement of antioxidant defense enzymes (Sarraf et al., 2022; Preetha et al., 2023; Bilge et al., 2025). Previous studies have demonstrated that metal NPs, varying in size, concentration, and surface charge, can markedly influence the growth and development of diverse plant species (Djanaguiraman et al., 2018; Faisal et al., 2024; Soni et al., 2026).

Cerium oxide nanoparticles (CeO_2_ NPs) have emerged as materials of significant interest in materials science, chemistry, physics, medicine, and agricultural research. Previous studies indicate that CeO_2_ NPs significantly enhance plant tolerance to abiotic stress through upregulation of the antioxidant defense system (Sharma et al., 2019; Mohammadi et al., 2021). The multi-enzyme capabilities of CeO_2_ NPs, including mimic properties of POD, CAT, and SOD (Hamidian and Sarani, 2025; Shiraz et al., 2025). CeO_2_ NPs minimize oxidative stress by effectively binding free radicals, including H_2_O_2_, ^1^O_2_, OH, and O_2_•^-^ (Xu and Qu, 2014). CeO_2_ NPs have been shown to affect plant development and yield, as well as alter the nutritional composition of plants, due to their ability to promote growth at optimal concentrations (Du et al., 2015). For instance, CeO_2_ NPs applied at 100 mg kg^-1^ improved photosynthesis, water use efficiency, and rubisco activity in soybean (Cao et al., 2017). CeO_2_ NPs have been reported to have similar beneficial effects on the growth of wheat (Rico et al., 2014), tomato yield (Wang et al., 2012), and *Helianthus annuus*. However, to date, no studies have investigated the role of CeO_2_ NPs in alleviating Cr stress in rice.

The novelty of this research lies in its comprehensive evaluation of the effects of CeO_2_ NPs on rice under Cr stress. Unlike previous studies, this investigation systematically examines physiological, biochemical, and metabolic profiling responses of CeO_2_ NPs (100 ppm), providing a deeper understanding of their functional roles. Application of CeO_2_ NPs (100 ppm) enhances Cr stress (100 µM) tolerance in rice by improving photosynthetic efficiency, biochemical and antioxidant defense systems, and metabolic homeostasis, thereby mitigating Cr-induced adverse effects. These integrative insights underscore the potential of CeO_2_ NPs as a targeted, nanotechnology-driven solution for enhancing Cr-resilience in rice.

## 2 Materials and methods

### 2.1 Plant material and growth conditions

**Figure 1:**
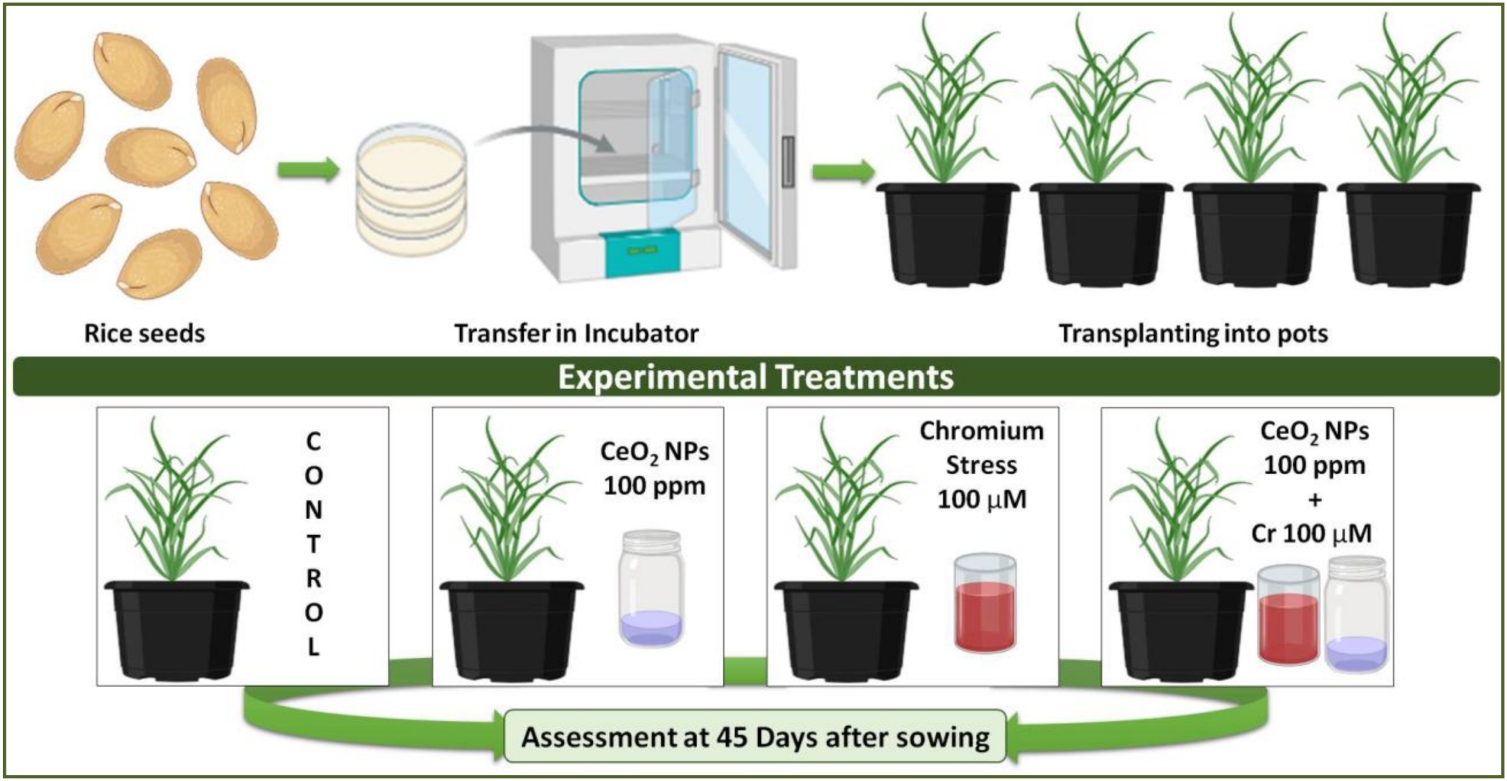
Schematic illustration of the experimental design showing seed treatment, growth conditions, and treatments used to assess growth and physiological responses of rice at 45 days after sowing.

Healthy seeds of *Oryza sativa* L. (rice) var. DDR Dhan 73 were surface-sterilized with 0.01% (w/v) sodium hypochlorite and rinsed 2–3 times with distilled water prior to sowing. The experiment comprised four treatments: control, cerium oxide nanoparticles (CeO_2_ NPs; 100 ppm), chromium stress (Cr; 100 µM), and combined CeO_2_ NPs (100 ppm) + Cr stress (100 µM). Cr stress and CeO_2_ NPs were applied at the seed stage by soaking seeds in their respective solutions for 12 h. For germination assessment, control and treated seeds were incubated at 25 °C in a growth incubator (Blue Pard MGCHP). After 15 days, uniformly germinated seedlings were transplanted into plastic pots containing Alfisol soil with 0.35% organic carbon, a cation exchange capacity of 15.5 cmol kg^-1^, 215 mg kg^-1^ total P, 60 mg kg^-1^ available N, 5.5 mg kg^-1^ available P, and 80 mg kg^-1^ available K. Plants were irrigated daily according to their respective treatment regimes and arranged in a completely randomized design with four replicates per treatment (n = 4). At 45 days after sowing (DAS), corresponding to the vegetative growth stage, plants were harvested for the evaluation of growth, physiological, and biochemical parameters (**Figure 1**).

### 2.2 Characterization of CeO_2_ NPs

**Figure 2:**
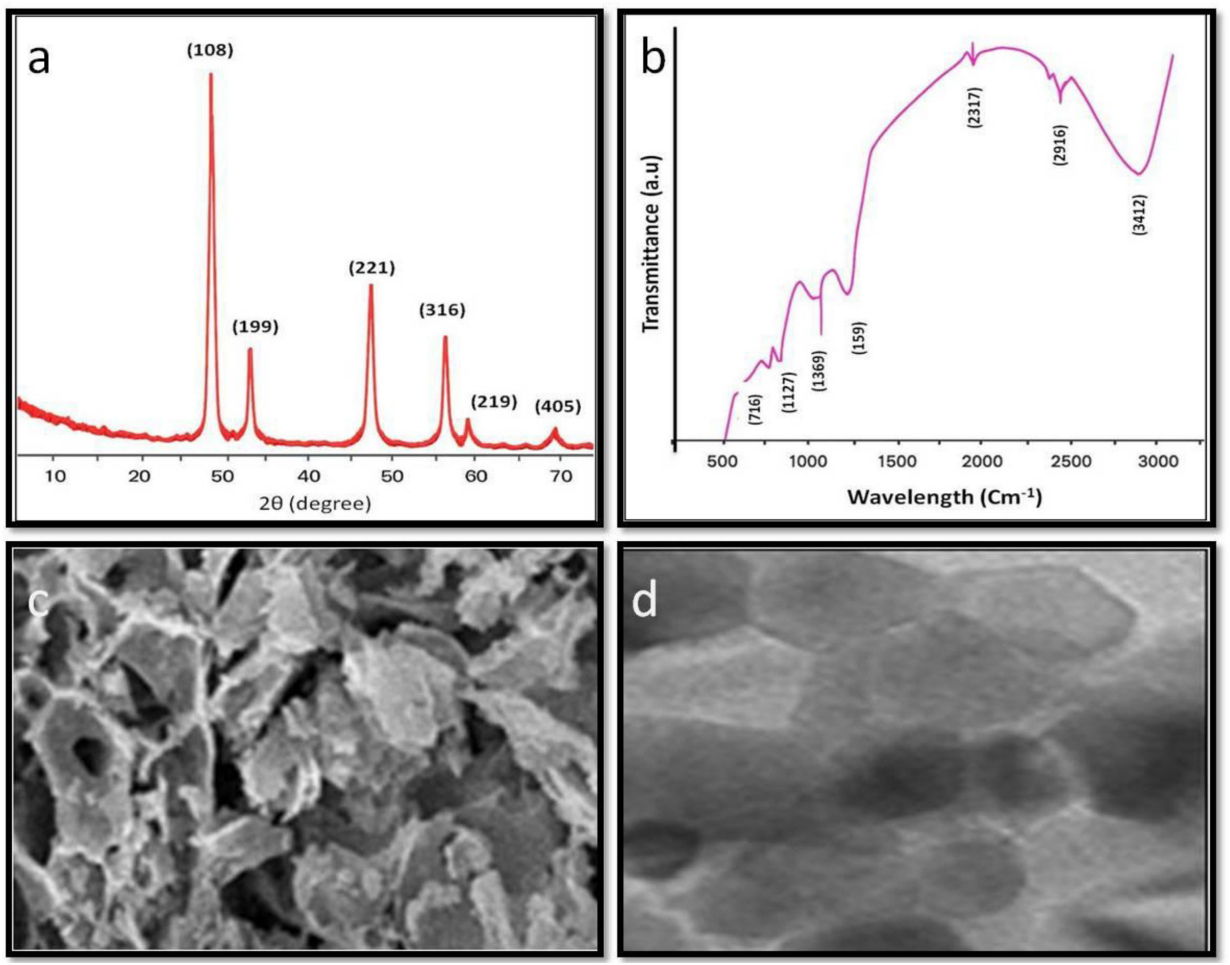
Characterization of cerium oxide nanoparticles (CeO_2_ NPs): **(a)** XRD pattern, **(b)** FTIR spectrum, **(c)** SEM micrograph, and **(d)** TEM image, illustrating the structural, functional, and morphological features of the CeO_2_ NPs.

The X-ray diffraction (XRD) patterns of CeO_2_ NPs were obtained using a Rigaku Smart Lab X-ray diffractometer over a 2θ range of 20°–80°, employing Cu Kα radiation (λ = 1.540 Å) operated at 40.0 kV and 30.0 mA. Fourier transform infrared (FTIR) spectra were recorded in the range of 450–4000 cm^-1^ using the KBr pellet method on a PerkinElmer FTIR spectrometer (Spectrum Two). The surface morphology of the CeO_2_ NPs was examined using a scanning electron microscope (SEM; JSM-6510LV, JEOL, Japan), with samples sputter-coated with gold prior to analysis. Transmission electron microscopy (TEM) analysis was performed using a TECNAI G20 HR-TEM to evaluate the morphology and particle size distribution of the CeO_2_ NPs (**Figure 2**).

### 2.3 Growth attributes

For the measurement of plant growth-associated traits, fully grown rice plants (45 DAS) were carefully uprooted and washed gently 2–3 time to remove the adhere soil particles. The plants shoot and root length was precisely measured with a meter scale, and these traits were correspondingly added to estimate the overall plant height. For dry weight determination, shoots and roots were oven-dried at 70 °C for 48 h until a constant weight was achieved and subsequently weighed using an electronic analytical balance.

### 2.4 Evaluation of photosynthetic and carbohydrate metabolism traits

Determination of photosynthetic traits, including total chlorophyll (Chl) content and ribulose-1,5-bisphosphate carboxylase/oxygenase (RuBisCO) activity, were estimated using the methods as provided in Barnes et al. (1992); Usuda (1985), respectively, using a double beam UV-visible spectrophotometer (Systronics, Model No. 2203). Carbon utilization-related traits, including total carbohydrates and non-structural carbohydrates (NSCs) such as sucrose, starch, and soluble sugars, were quantified following the protocols described by Dubois et al. (1951); Hedge et al. (1962); Dawalibi et al. (2015); Liu et al. (2018), respectively, with minor modifications. Further, C-metabolizing enzymes, including sucrose synthase (SuSy), adenosine diphosphate-glucose pyrophosphorylase (ADPG-Ppase), amylase activities were assayed using the protocols as illustrated in Morell and Copeland (1985); Xie et al. (2016); Bernfeld (1955); Huang et al. (2013), respectively, with few moderations.

### 2.5 Assessment of gas exchange attributes

A portable photosynthesis system (model LI-6400; LI-COR, Lincoln, NE, USA) was used to measure various photosynthetic parameters, including the net photosynthetic rate (*Pn*), stomatal conductance (*gs*), intercellular CO_2_ concentration (*Ci*), and transpiration rate (*E*). During the measurements, the environmental conditions inside the leaf chamber were maintained constant, with an ambient temperature of 25± 2 °C, relative humidity of 85 ± 5%, CO_2_ concentration of 600 µmol mol^−1^, and a photosynthetic photon flux density (PPFD) of 800 µmol m^−2^ s^−1^ provided by a red/blue LED light source.

### 2.6 Assessment of oxidative stress indicators

Oxidative stress markers, including thiobarbituric acid reactive substances (TBARS), hydrogen peroxide (H_2_O_2_), superoxide anion (O_2_•^-^), and methylglyoxal (MG), were quantified according to the methods described by Heath and Packer (1968); Okuda et al. (1991); Mohammadi and Karr (2001); Wild et al. (2012), respectively, using a double-beam UV-visible spectrophotometer in leaf tissues.

### 2.7 Assessment of enzymatic and non-enzymatic scavengers or antioxidants

The activities of enzymatic antioxidants, including SOD, ascorbate peroxidase (APX), glutathione peroxidase (GPX), monodehydroascorbate reductase (MDHAR), dehydroascorbate reductase (DHAR), glutathione reductase (GR), glutathione S-transferase (GST), glyoxalase I (Gly-I), and glyoxalase II (Gly-II), were assayed following the procedures described by Khan et al. (2020) using a double-beam UV-visible spectrophotometer. In addition, the levels of non-enzymatic antioxidants, namely ascorbate (AsA) and glutathione (GSH), were quantified according to the methods of Masato (1980); Griffith (1980), respectively, using the same instrument.

### 2.8 Assessment of nitric oxide biosynthesis-associated traits

Nitric oxide (NO) content was quantified following the method described by Zhou et al. (2005) using a double-beam UV-visible spectrophotometer. In addition, nitric oxide synthase (NOS) activity was assayed according to the protocol of Zhao et al. (2009) with minor modifications. Furthermore, the qualitative detection of NO in root tips was carried out following the method described by Desikan et al. (2002) with slight modifications.

### 2.9 Estimation of secondary metabolites

Secondary metabolites, including phenol and flavonoid contents, were determined by undertaking the protocols as mentioned in Chun et al. (2003); Zhishen et al. (1999), respectively, using a double beam UV-visible spectrophotometer.

### 2.10 Estimation of cellulose and lignin content

The root cellular defense-associated parameters, including cellulose and lignin contents, were determined through adopting the protocols as demonstrated in Updegraff (1969); Hussain et al. (2021), respectively, using a double beam UV-visible spectrophotometer.

### 2.11 Evaluation of ionic homeostasis and K^+^/Na^+^ traits

Ionic homeostasis, in terms of sodium (Na^+^) and potassium (K^+^) contents, was determined using a flame photometer (Systronics, Model 128) following the protocols described by Munns et al. (2010); Walker et al. (1995), respectively. Based on these measurements, the K^+^/Na^+^ ratios in root and shoot tissues were calculated.

### 2.12 Statistical analysis

Two-way analysis of variance (ANOVA) has been used to analyze the statistical data using RStudio (version 2022.02.3–492). The bar graphs, principal component analysis (PCA), heat map, correlation and multiple regression plots were also generated using RStudio (version 2022.02.3–492). Data were presented as treatment means ± SE (n = 4). Data followed by the same letter did not significantly differ according to Tukey’s Honest Significant Difference (HSD) test at 0.001<p<0.01.

## 3 Results

### 3.1 Effect of CeO_2_ NPs on the growth of rice plants under Cr stress

**Figure 3:**
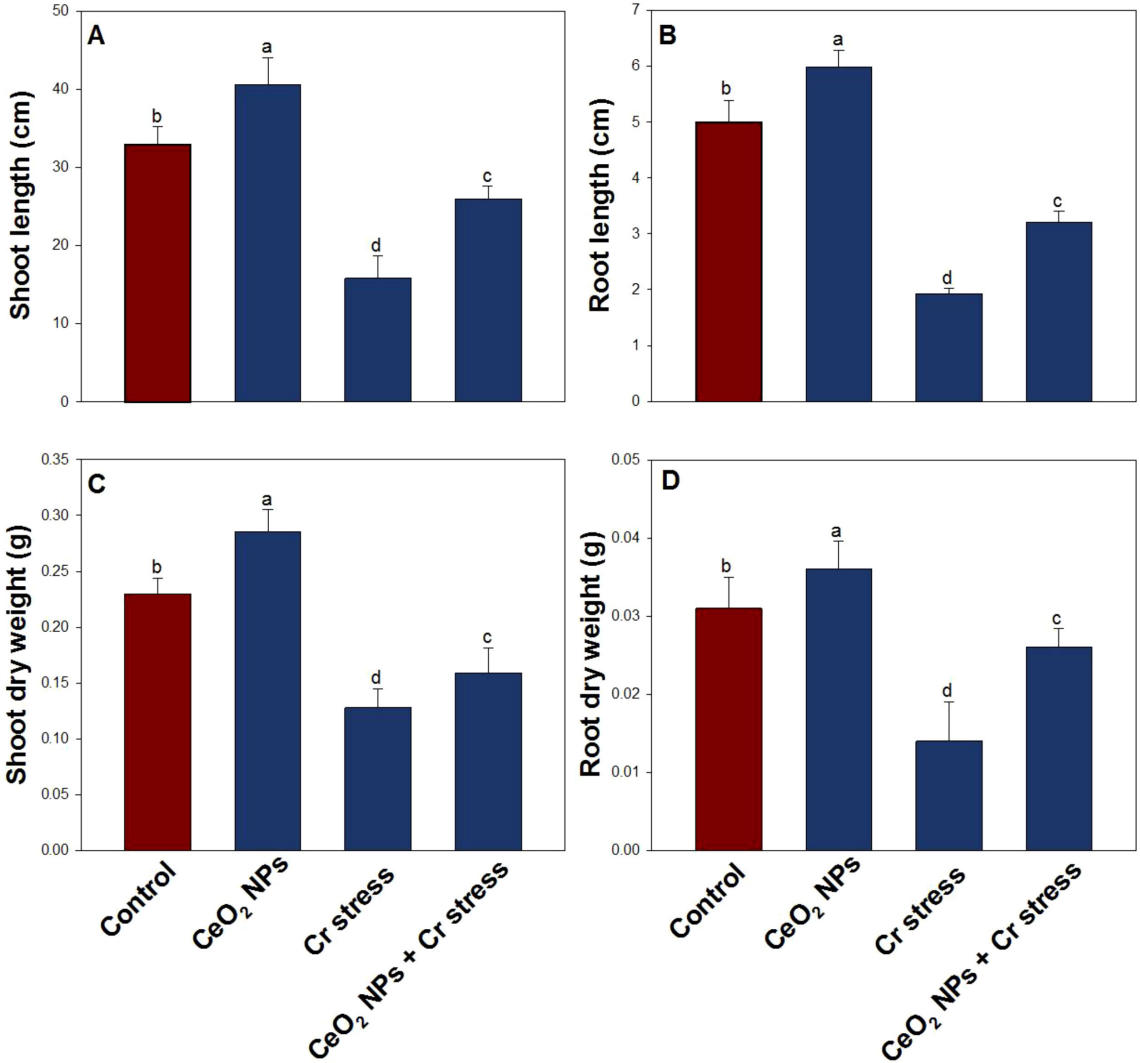
Growth indices in rice exposed to CeO_2_ NPs under Cr stress (45 DAS). **(A)** Shoot length. **(B)** Root length. **(C)** Shoot dry weight. **(D)** Root dry weight. Bars represent the mean of 4 experimental replicates. Distinct letters on the graphs indicate significant differences between the control and treatment groups at P<0.05, as determined by Tukey’s test.

**Figure 4:**
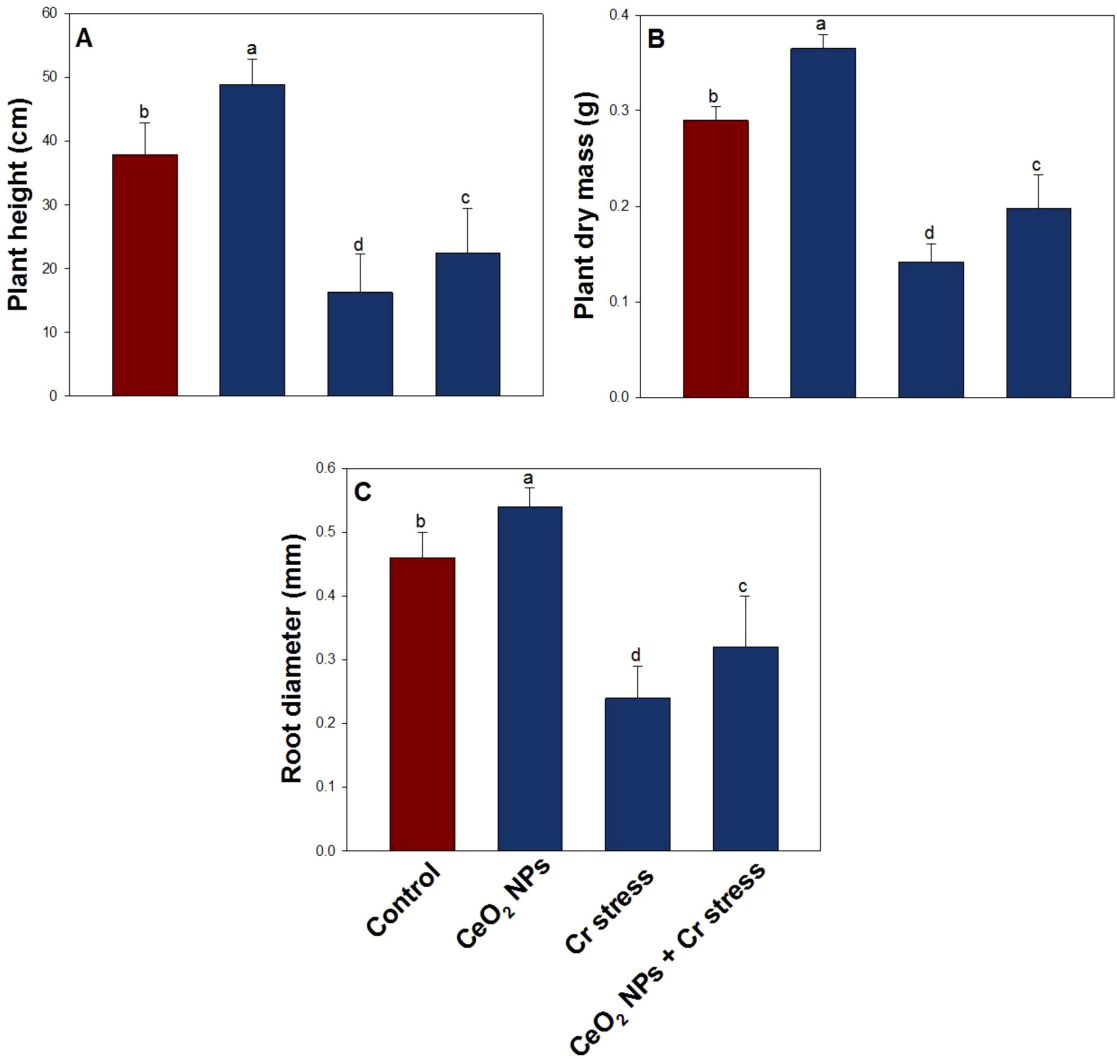
Growth indices in rice exposed to CeO_2_ NPs under Cr stress (45 DAS). **(A)** Plant height. **(B)** Plant dry mass. **(C)** Root diameter. Bars represent the mean of 4 experimental replicates. Distinct letters on the graphs indicate significant differences between the control and treatment groups at P<0.05, as determined by Tukey’s test.

Chromium has a negative impact on all growth indices (**Figures 3**, **4**). The decreased in root length by 58.4%, shoot length by 51.7%, root dry weight by 53.1%, shoot dry weight by 44.9%, plant height by 57.3%, plant dry mass by 51.8%, and root diameter by 47.5%, when treated with Cr (100 µM) compared to the control. However, when treated with Cr combined with CeO_2_ NPs, the root length, shoot length, root dry weight, shoot dry weight, plant height, plant dry mass, and root diameter increased significantly by 31.2%, 37.9%, 38.4%, 33.6%, 38.7%, 40.2%, and 36.4%, respectively, compared to only Cr-stressed plants.

### 3.2 Effect of CeO_2_ NPs on photosynthesis and carbohydrate metabolism in rice plants under Cr stress

Under Cr stress, photosynthetic performance and carbohydrate metabolism were markedly altered (**Table 1**). Chromium stress reduced chlorophyll content by 67.5%, RuBisCO by 69.1%, total carbohydrate by 38.5% and sucrose by 53.6%, starch by 51.4%, sucrose synthase by 49.5%, ADPG-Ppase by 69.3%, and amylase by 68.2%, with their respective control plants. However, application of CeO_2_ NPs increased all the parameters by 31.7% (chlorophyll content), 36.1% (RuBisCO), 21.8% (total carbohydrate), 23.5% (sucrose), 24.8% (starch), sucrose synthase, ADPG-Ppase, and amylase also follow the same trends. In Cr-stressed rice plants, CeO_2_ NPs partially recovered the activity of all the aforesaid parameters.

**Table 1:** Total chlorophyll (Chl; g kg^−1^ FM) content, ribulose-1, 5-bisphosphate carboxylase/oxygenase (RuBisCO; μmol CO_2_ mg^−1^ protein min^−1^) activity, carbohydrate content (mg g^−1^ DW), sucrose content (mg g^−1^ DW), starch content (mg g^−1^ DW), sucrose synthase activity (U mg^−1^ protein), adenosine diphosphate (ADP)-glucose pyrophosphorylase (ADPG-Ppase) activity (μmol s^−1^ g^−1^ protein), amylase activity (U mg^−1^ protein), in the leaves of rice exposed to Cr stress at 45 DAS, and supplementation of CeO_2_ NPs (100 ppm) has been administered through seed priming.

Parameters	Control	CeO-NPs	Cr stress	Cr stress + CeO-NPs
Chl	5.14 ± 0.02b	6.73 ± 0.04a	1.59 ± 0.03 d	2.28 ± 0.05c
RuBisCO	18.64 ± 0.23b	25.35 ± 0.09a	6.15 ± 0.03d	10.39 ± 0.03c
Carbohydrate	1.25 ± 0.03b	1.51 ± 0.04a	0.775 ± 0.01d	0.968 ± 0.014c
Sucrose	18.88 ± 0.09b	23.22 ± 0.08a	8.87 ± 0.04d	11.53 ± 0.05c
Starch	2.31 ± 0.06b	2.86 ± 0.04a	1.13 ± 0.05d	1.51 ± 0.07c
Sucrose synthase	17.68 ± 0.06b	21.92 ± 0.03a	9.01 ± 0.11d	12.71 ± 0.06c
ADPG-Ppase	4.25 ± 0.05b	5.27 ± 0.04a	1.31 ± 0.04d	1.996 ± 0.01c
Amylase	1.81 ± 0.06b	2.42 ± 0.03a	0.579 ± 0.01d	0.897 ± 0.01c

Analyses were conducted at 45 DAS with data presented as treatments mean± SE (n = 4). Data followed by the same letter do not significantly differ by Tukey’s test.

### 3.3 Effect of CeO_2_ NPs on leaf gas exchange parameters in rice plants under Cr stress

**Figure 5:**
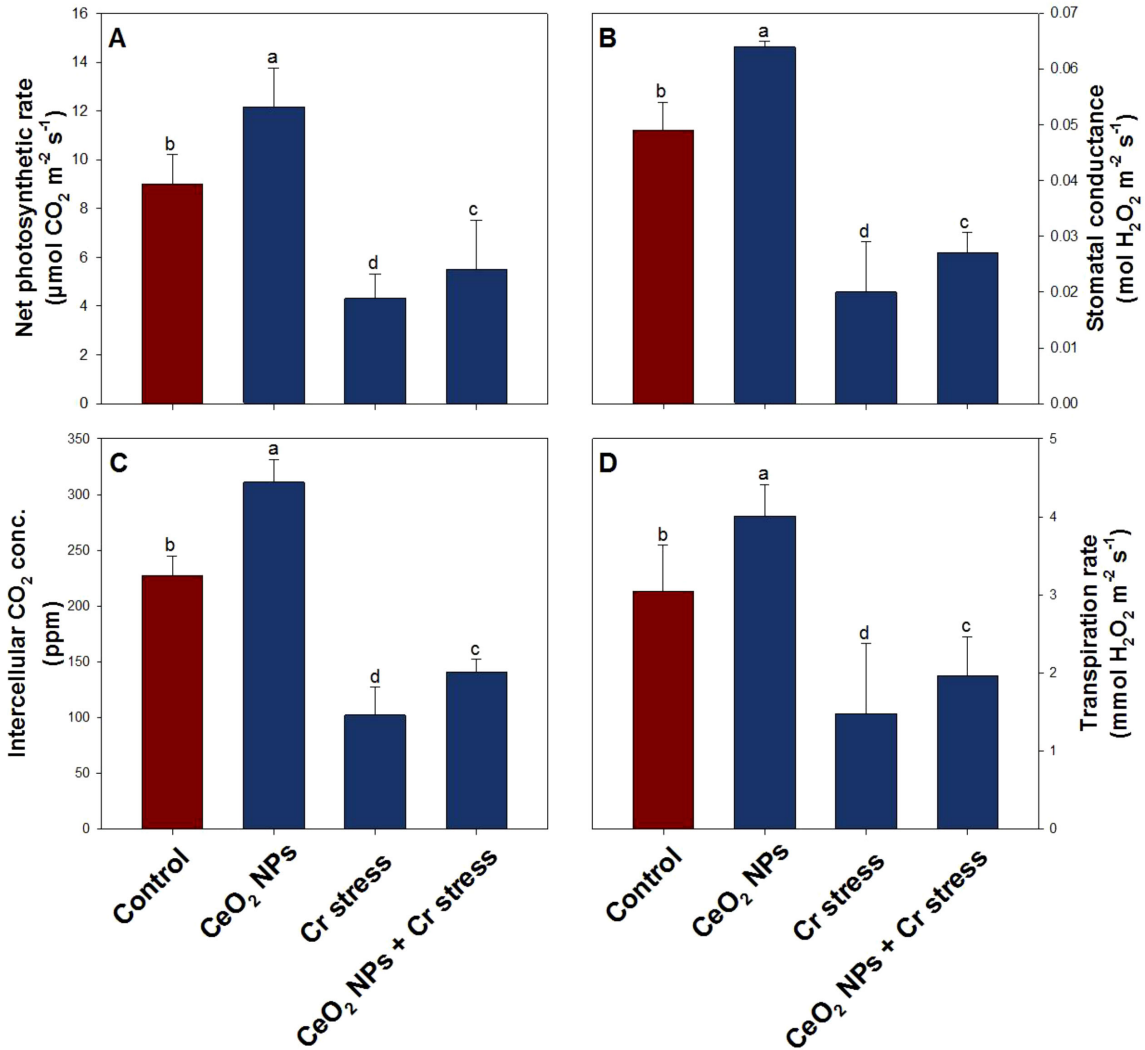
Leaf gas exchange parameters in rice exposed to CeO_2_ NPs under Cr stress (45 DAS). **(A)** Net photosynthetic rate. **(B)** Stomatal conductance. **(C)** Intercellular CO_2_ conc. **(D)** Transpiration rate. Bars represent the mean of 4 experimental replicates. Distinct letters on the graphs indicate significant differences between the control and treatment groups at P<0.05, as determined by Tukey’s test.

Chromium stress caused a pronounced decline in gas exchange, reducing *Pn* by 52%, *gs* by 58%, *Ci* by 55%, and *E* by 51% relative to the control (**Figure 5**). However, CeO_2_ NPs treatment significantly improved photosynthetic performance, as evidenced by increases of 35% in *Pn*, 31% in *gs*, 37% in *Ci*, and 32% in *E* compared with the control. Notably, the ameliorative treatment under Cr-stress partially restored photosynthetic activity, with *Pn*, *gs*, *Ci*, and *E* increasing by 28-39% over the stressed plants (**Figure 5**).

### 3.4 Effect of CeO_2_ NPs on oxidative stress indicators in rice plants under Cr stress

**Figure 6:**
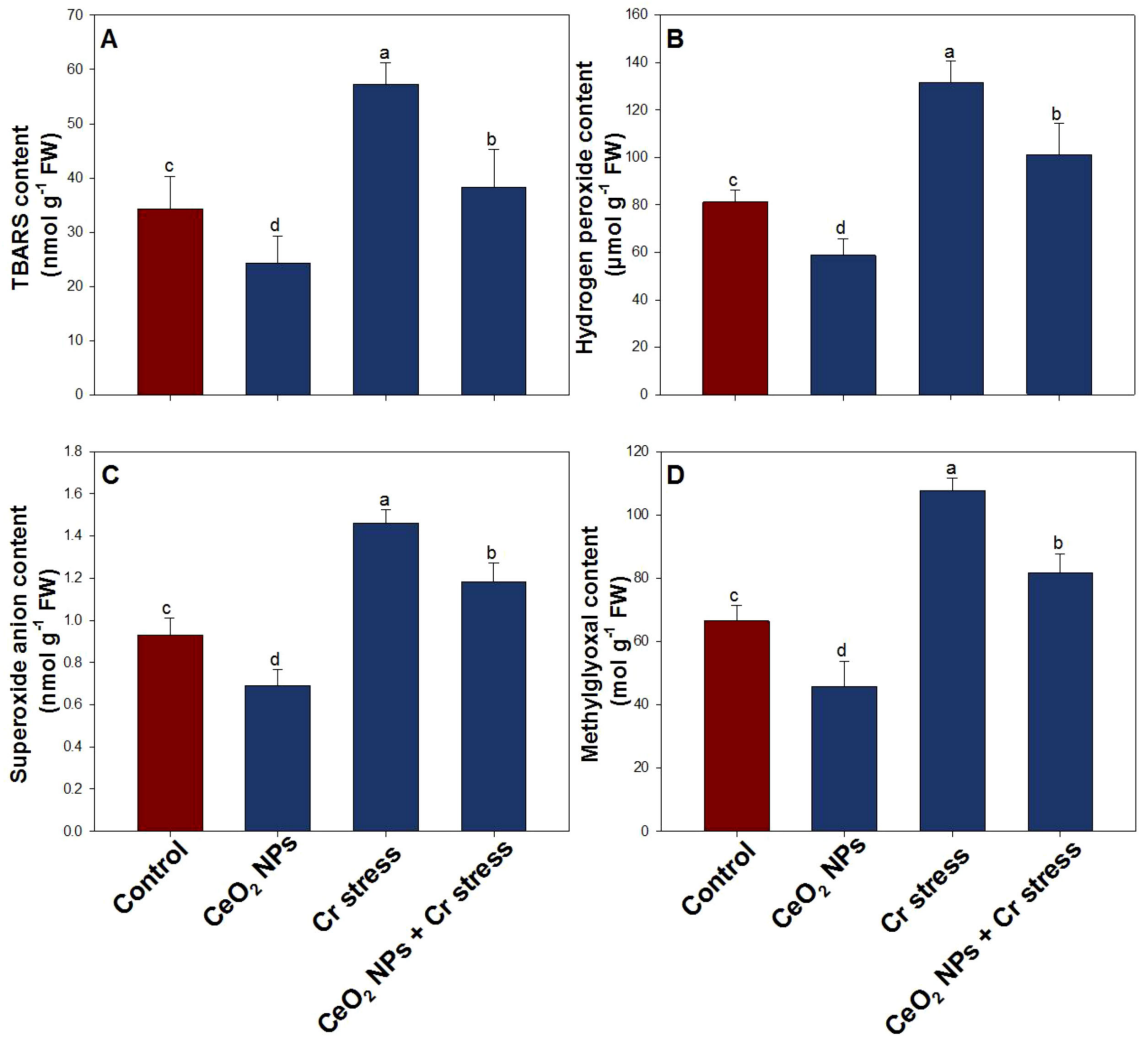
Oxidative stress indicators in rice exposed to CeO_2_ NPs under Cr stress (45 DAS). **(A)** TBARS content. **(B)** Hydrogen peroxide content. **(C)** Superoxide anion content. **(D)** Methylglyoxal content. Bars represent the mean of 4 experimental replicates. Distinct letters on the graphs indicate significant differences between the control and treatment groups at P<0.05, as determined by Tukey’s test.

The oxidative stress indicators exhibited substantial variation among treatments, reflecting differences in cellular redox status. Chromium stress markedly intensified oxidative stress, as evidenced by pronounced increases in TBARS by 67.2%, H_2_O_2_ by 61.9%, O_2_^·-^ by 58.7%, and methylglyoxal 62.8% relative to the control plants (**Figure 6**). The treatment of CeO_2_ NPs significantly reduced oxidative damage, leading to decreases in all parameters, with TBARS by 29.4%, H_2_O_2_ by 27.6%, O_2_^·-^ by 25.8%, and methylglyoxal contents by 31.3%, respectively, compare to control plants. Notably, in the presence of Cr stress, CeO_2_ NPs protect rice plants by increasing all the above stress indicators by 21.4% (TBARS), 23.1% (H_2_O_2_), 19.6% (O_2_^·-^), and 24.9% (methylglyoxal contents), in respects to Cr-stressed plants.

### 3.5 Effect of CeO_2_ NPs on antioxidant enzymes activity in rice plants under Cr stress

The activities of antioxidant enzymes, redox metabolites, and glyoxalase system components showed a marked and progressive enhancement across treatments compared with the control (**Table 2**). SOD activity increased from 24.8%, 54.0%, and 86.8% in successive treatments, indicating a strong activation of the primary ROS-scavenging system. Similarly, APX activity rose significantly from 24.32%, 58.1%, and 106.5%, while GPX showed an increase of 35.85%, 64.18%, and 113.51% over control values in CeO_2_ NPs, Cr stress and Cr stress + CeO_2_ NPs, respectively. Enzymes associated with the ascorbate–glutathione cycle were substantially upregulated. Monodehydroascorbate reductase activity increased by 26.45%, 68.31%, and 102%, whereas DHAR showed increments of 18.34%, 31.0%, and 91.1% relative to the control. Glutathione reductase exhibited a pronounced enhancement, increasing from 8.89 in the control to 39.23%, 76.28%, and 128.5%. Likewise, GST activity increased progressively by 22.85%, 72.39%, and 116.8%. Non-enzymatic antioxidants also followed a similar trend. Ascorbic acid content increased from 27.12%, 51.26%, and 118.37%, while GSH content rose from 17.36%-87.33%. The glyoxalase system was significantly stimulated, as evidenced by increases in Gly-I activity from 18.6%, 48.2%, and 82.3%, and Gly-II from 31.42%, 73.61%, and 103.43%. Overall, the coordinated upregulation of antioxidant enzymes, redox metabolites, and glyoxalase components demonstrates a strengthened cellular defense mechanism under the applied treatments (**Table 2**).

**Table 2:** SOD (U mg^-1^ protein min^-1^), APX (U mg^-1^ protein min^-1^), GPX (U mg^-1^ protein min^−1^), MDHAR (U mg^-1^ protein min^-1^), DHAR (U mg^-1^ protein min^-1^), GR (U mg^-1^ protein min^-1^), GST (U mg^-1^ protein min^-1^), AsA (mg g^-1^ FW), GSH (nmol g^-1^ FW), Gly-I/II (μmol min^-1^ mg^-1^ protein) in the leaves of rice exposed to Cr stress, and supplementation of CeO_2_ NPs.

Parameters	Control	CeO-NPs	Cr stress	Cr stress + CeO-NPs
SOD	33.19 ± 3.12d	41.42 ± 2.01c	51.16 ± 1.98b	61.99 ± 1.64a
APX	4.98 ± 0.61d	6.19 ± 0.30c	7.87 ± 0.19b	10.28 ± 0.39a
GPX	0.041 ± 0.003d	0.055 ± 0.001c	0.067± 0.006b	0.087 ± 0.0005a
MDHAR	53.28 ± 1.18d	67.33 ± 3.62c	89.62 ± 4.68b	107.62 ± 1.53a
DHAR	76.65 ± 1.28d	90.70 ± 4.07c	114.44 ± 6.98b	146.47 ± 2.89a
GR	8.89 ± 1.24d	12.37 ± 1.04c	15.67 ± 2.18b	20.31 ± 2.20a
GST	31.05 ± 2.42d	38.14 ± 1.73c	53.52 ± 2.37b	67.32 ± 1.36a
AsA	0.68 ± 0.01d	0.86 ± 0.008c	1.17 ± 0.048b	1.48 ± 0.040a
GSH	184 ± 3.03d	215.94 ± 16.8c	278.90 ± 5.40b	344 ± 10.25a
Gly-I	0.31 ± 0.02d	0.367 ± 0.01c	0.459 ± 0.012b	0.560 ± 0.006a
Gly-II	30.99 ± 2.61d	40.72 ± 3.47c	53.80 ± 2.32b	63.03 ± 2.47a

Analyses were conducted at 45 DAS with data presented as treatments mean± SE (n = 4). Data followed by the same letter do not significantly differ by Tukey’s test.

### 3.6 Effect of CeO_2_ NPs on NO signaling and secondary metabolites in rice plants under Cr stress

**Figure 7:**
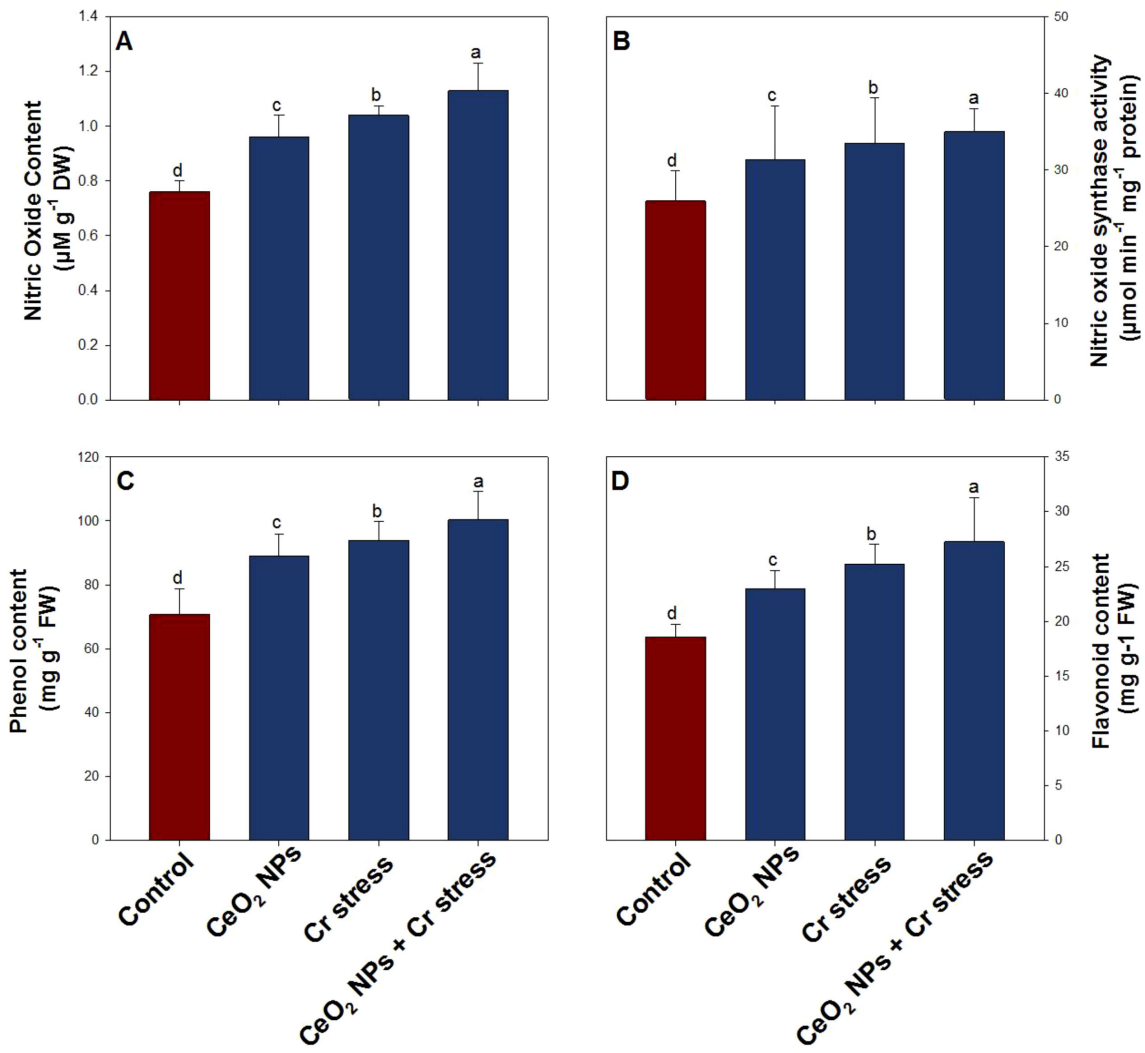
Nitric oxide signaling and secondary metabolites in rice exposed to CeO_2_ NPs under Cr stress (45 DAS). **(A)** Nitric oxide content. **(B)** Nitric oxide synthase activity. **(C)** Phenol content. **(D)** Flavonoid content. Bars represent the mean of 4 experimental replicates. Distinct letters on the graphs indicate significant differences between the control and treatment groups at P<0.05, as determined by Tukey’s test.

Nitric oxide (NO) metabolism and secondary metabolite accumulation were markedly influenced by the treatments. In the absence of CeO_2_ NPs (100 ppm), Cr stress causes a modest increase in NO content by 38.4%, NOS activity by 29.1%, phenol content by 33.6%, and flavonoid content by 36.7%, compare to control plants (**Figure 7**). Adding CeO_2_ NPs to Cr-stressed plants improve NO signaling and secondary metabolites and the highest values were recorded, which elevated NO content by 49.6%, NOS activity by 35.7%, phenol content by 42.3%, and flavonoid content by 47.9% relative to the control.

### 3.7 Effect of CeO_2_ NPs on root carbon fractions in rice plants under Cr stress

**Figure 8:**
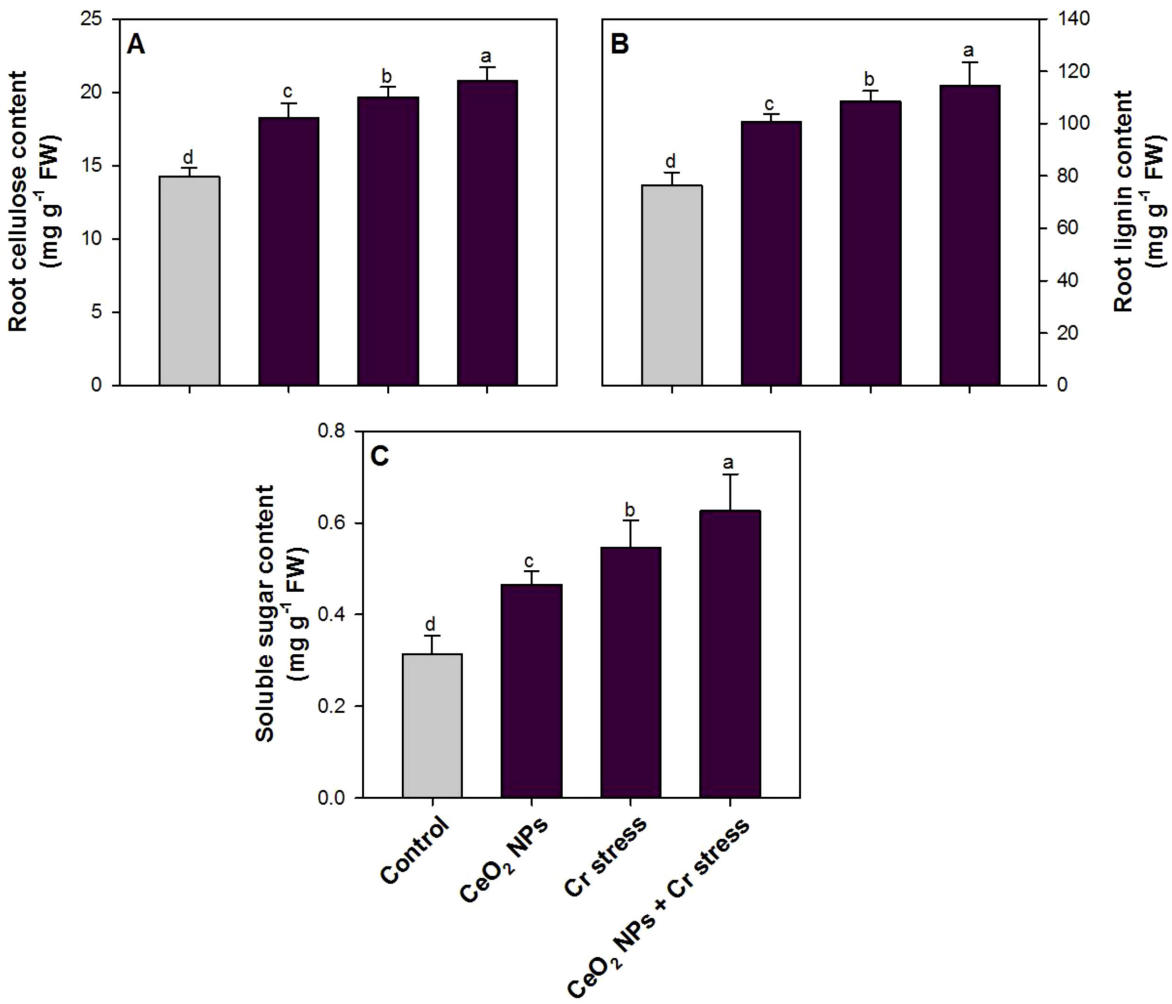
Root carbon fractions in rice exposed to CeO_2_ NPs under Cr stress (45 DAS). **(A)** Root cellulose content. **(B)** Root lignin content. **(C)** Soluble sugar content. Bars represent the mean of 4 experimental replicates. Distinct letters on the graphs indicate significant differences between the control and treatment groups at P<0.05, as determined by Tukey’s test.

The content of root cellulose, lignin, and soluble sugar showed a marked increase across treatments compared with the control (**Figure 8**). Chromium stress increased root cellulose content by 38.3%, lignin content by 42.7%, and soluble sugar content by 76.5% compared to control plants (**Figure 4**). Application of CeO_2_ NPs, alone as well as in combination increased all the mentioned parameters by 28.2% and 46.1% (root cellulose), 31.6% and 50.8% (lignin), and 50.5% and 102.5% (soluble sugar) compare to control plants (**Figure 8**).

### 3.8 Effect of CeO_2_ NPs on ionic homeostasis and K^+^/Na^+^ balance in rice plants under Cr stress

**Figure 9:**
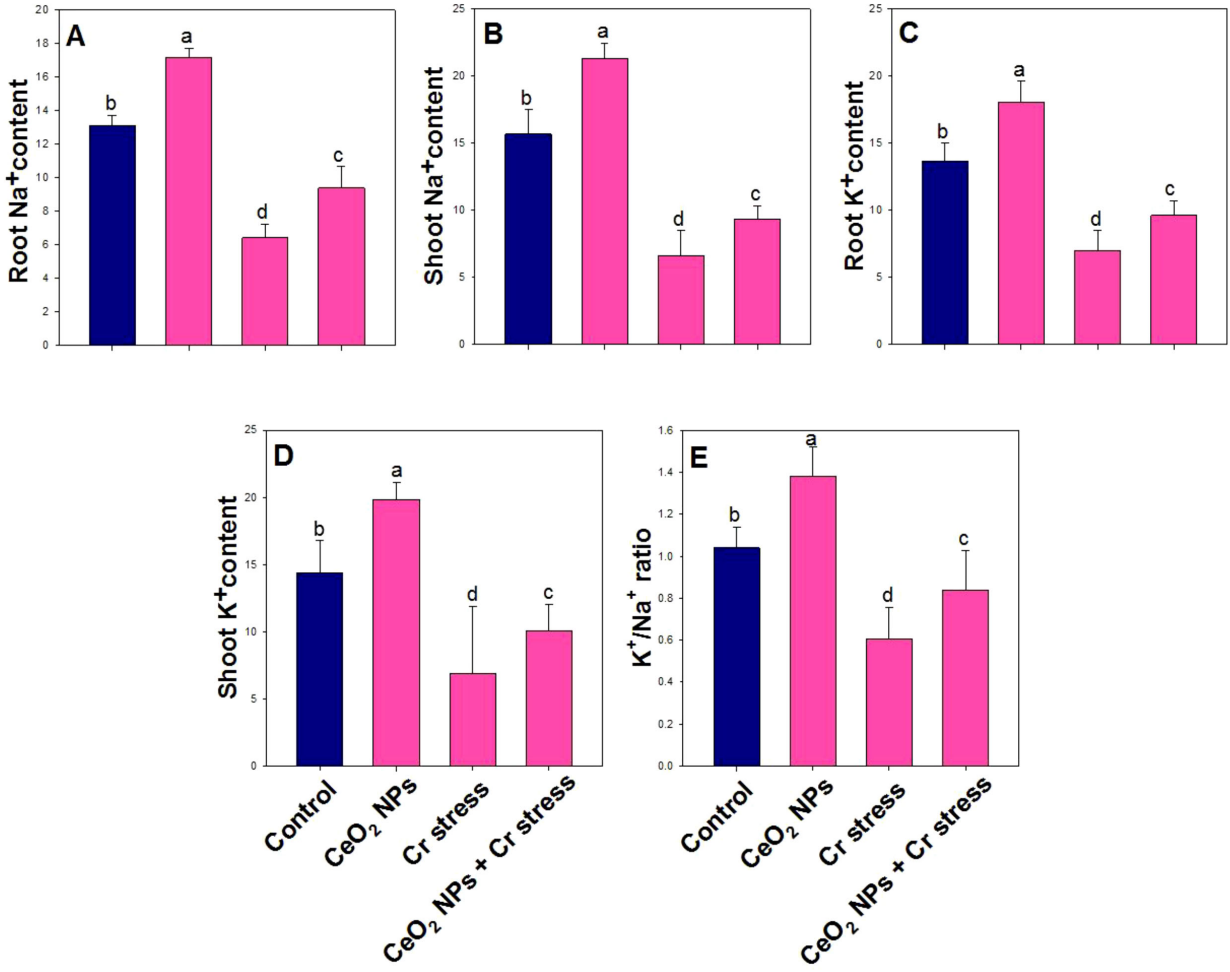
Ionic homeostasis and K^+^/Na^+^ balance in rice exposed to CeO_2_ NPs under Cr stress (45 DAS). **(A)** Root Na^+^ content. **(B)** Shoot Na^+^ content. **(C)** Root K^+^ content. **(D)** Shoot K^+^ content. **(E)** K^+^/Na^+^ content. Bars represent the mean of 4 experimental replicates. Distinct letters on the graphs indicate significant differences between the control and treatment groups at P<0.05, as determined by Tukey’s test.

Ion homeostasis was significantly affected by the different treatments, as reflected in the concentrations of Na^+^ and K^+^ in roots and shoots and the resulting K^+^/Na^+^ ratio. Chromium stress severely disrupted ionic balance, leading to pronounced reductions in root and shoot Na^+^ (51% and 58%) and K^+^ (49% and 52%), accompanied by a sharp decline in the K^+^/Na^+^ ratio to 42% decrease compare to control plants (**Figure 9**). Notably, the ameliorative treatment under Cr-stress significantly restored ionic homeostasis, elevating root and shoot Na^+^ by 46% and 42% and K^+^ by 38% and 46% and increasing the K^+^/Na^+^ ratio to 39% relative to Cr-stressed plants.

## 4 Discussion

In this study, for the first time, we showed that the application of CeO_2_ NPs could alleviate Cr-induced toxicity in the rice plants at an optimal concentration of Cr (100 µM). Application of CeO_2_ NPs markedly alleviated Cr-triggered oxidative stress by enhancing antioxidant enzyme activities and restoring photosynthetic performance through increased chlorophyll accumulation. Chromium toxicity is known to severely impair plant morphological, physiological, and biochemical processes by disrupting ionic homeostasis, carbon metabolism, nitric oxide signaling, secondary metabolite biosynthesis, stomatal conductance, and overall photosynthetic efficiency (Ertani et al., 2017; Zhiguo et al., 2022). Consistent with these reports, Cr exposure in the present study caused pronounced reductions in root and shoot length, dry biomass, plant height, total plant dry mass, and root diameter, reflecting substantial inhibition of both below- and above-ground growth. Such growth suppression under Cr stress is primarily attributed to restricted cell division and elongation, impaired nutrient and water uptake, inhibition of photosynthesis, and excessive generation of ROS that damage cellular structures and metabolic pathways (Shanker et al., 2005). Notably, CeO_2_ NPs application effectively counteracted these detrimental effects, resulting in a partial but significant restoration of growth attributes, with overall improvements of 31–40% across all measured traits. These findings align with earlier studies reporting NP-mediated growth enhancement under stress conditions, such as TiO_2_ NP-induced improvement of agronomic traits in *Moldavian balm* (Gohari et al., 2020). The high surface area and nanoscale dimensions of NPs facilitate their penetration into plant tissues, enabling mitigation of metal-induced toxicity and stimulation of growth processes (Singh et al., 2019), often through improved water-use efficiency, stomatal conductance, transpiration, and photosynthetic capacity (Hezaveh et al., 2019). Mechanistically, CeO_2_ NPs alleviate Cr toxicity through their unique Ce^3+^/Ce^4+^ redox cycling, which restores cellular redox balance, strengthens antioxidant defenses, maintains ionic and water homeostasis, and protects the photosynthetic apparatus. Furthermore, enhanced root architecture and increased root diameter under CeO_2_ NP treatment improve nutrient acquisition and plant anchorage, collectively contributing to greater assimilate production, biomass accumulation, and overall plant vigor. Taken together, these results underscore the potential of CeO_2_ NP as a promising nano-enabled strategy to enhance rice growth and resilience in chromium-contaminated environments.

Metal stress is well known to significantly reduce total chlorophyll content, thereby disrupting the photosynthetic apparatus and limiting photosynthetic efficiency (Chandra and Kang, 2016; Sytar et al., 2019). The decline in photosynthesis under heavy metal exposure is widely regarded as a hallmark of oxidative damage, resulting from pigment degradation, impaired light harvesting, and damage to chloroplast structure and function (Ashfaque et al., 2017; Mu et al., 2023). In the present study, Cr stress severely constrained photosynthetic performance and carbon metabolism in rice plants, as evidenced by marked reductions in chlorophyll content, RuBisCO activity, carbohydrate pools (total carbohydrates, sucrose, and starch), and the activities of key carbon-metabolizing enzymes. These pronounced declines reflect Cr-induced damage to chloroplast ultrastructure, inhibition of photosynthetic electron transport, and restricted carbon fixation, ultimately limiting assimilate production and energy availability (Panda and Choudhury, 2005; Gill and Tuteja, 2010). The strong suppression of RuBisCO and ADP-glucose pyrophosphorylase activities indicates impaired CO_2_ assimilation and starch biosynthesis, while reduced sucrose synthase and amylase activities suggest disrupted carbon partitioning and photo assimilate mobilization under metal toxicity. Notably, application of CeO_2_ NPs effectively restored chlorophyll levels and partially reversed Cr-induced photosynthetic inhibition. CeO_2_ NP have been shown to stimulate the synthesis of chlorophyll a, chlorophyll b, and carotenoids (Kataria et al., 2019; Etesami et al., 2021), and foliar application of CeO_2_ NP enhanced photosynthetic pigments in *Calendula officinalis* (Jahani et al., 2019). These beneficial effects are often attributed to increased micronutrient availability, reduced metal bioavailability, and enhanced tolerance to metal-induced oxidative stress (Rizwan et al., 2019; Etesami et al., 2021; Zhongyi et al., 2023). Supporting this mechanism, TiO_2_ NPs have been reported to enhance photosynthetic efficiency in soybean under Cd stress by penetrating chloroplasts and improving light absorption and electron transport (Singh and Lee, 2016). Mechanistically, CeO_2_ NP likely protect chloroplast membranes and the photosynthetic machinery through their unique Ce^3+^/Ce^4+^ redox cycling, which enables efficient scavenging of excess ROS and preservation of chlorophyll stability and enzymatic activity (Celardo et al., 2011). The recovery of carbohydrate content and carbon-metabolizing enzyme activities under CeO_2_ NP supplementation suggests improved photosynthate production, enhanced carbon flux toward sucrose and starch synthesis, and better cellular energy homeostasis under Cr stress. In parallel, Cr toxicity markedly impaired leaf gas exchange in rice plants, as reflected by substantial reductions in *Pn, gs, Ci, and E*, indicating the presence of both stomatal and non-stomatal limitations to photosynthesis. These declines are commonly associated with oxidative damage to guard cells, loss of membrane integrity, and inhibition of photosynthetic metabolism, which collectively restrict CO_2_ diffusion and assimilation (Panda and Choudhury, 2005; Gill et al., 2015). In contrast, CeO_2_ NP application significantly improved gas exchange parameters under non-stress conditions and partially restored them in Cr-stressed plants. By mitigating ROS accumulation in guard cells via Ce^3+^/Ce^4+^ redox cycling, CeO_2_ NP likely preserve stomatal functionality, maintain turgor regulation, and stabilize stomatal aperture. Enhanced *gs* facilitates greater CO_2_ influx, reflected by increased *Ci*, which in turn supports higher *Pn*, while recovery of transpiration indicates improved water relations and membrane stability.

Increasing Cr concentrations are known to suppress antioxidant enzyme activities, thereby exacerbating oxidative stress and disrupting cellular redox homeostasis (Bhaduri and Fulekar, 2012). Excessive generation of ROS under Cr stress overwhelms the antioxidant defense system, leading to lipid peroxidation, protein oxidation, membrane damage, and impairment of cellular structure and function (Ahmad et al., 2019; Ghori et al., 2019). Elevated levels of H_2_O_2_ and O_2_·^-^, together with increased TBARS, serve as key indicators of Cr-induced oxidative injury and chlorophyll degradation (Mallhi et al., 2019; Berni et al., 2019). In addition to oxidative stress, Cr toxicity also promotes the accumulation of MG, a highly cytotoxic carbonyl compound arising from stress-impaired carbohydrate metabolism, thereby triggering a coupled oxidative–carbonyl stress cascade that severely compromises cellular integrity and metabolic stability (Shanker et al., 2005; Hasanuzzaman et al., 2017; Alam et al., 2025). In the present study, application of CeO_2_ NP significantly enhanced the activities of key antioxidant enzymes, including SOD and APX, resulting in a pronounced reduction in ROS accumulation and lipid peroxidation under Cr stress. Antioxidant enzymes play a central role in detoxifying ROS and maintaining redox equilibrium (Kohli et al., 2017), and our findings are consistent with earlier reports demonstrating nanoparticle-mediated reinforcement of antioxidant defense systems under metal-induced oxidative stress (Hussain et al., 2019; Sun et al., 2019). Notably, CeO_2_ NP treatment led to marked declines in H_2_O_2_, O_2_·^-^, TBARS, and MG contents compared with Cr-stressed plants alone, indicating effective mitigation of both oxidative and carbonyl stress components. Mechanistically, CeO_2_ NP confer Cr tolerance through their unique and reversible Ce^3+^/Ce^4+^ redox cycling, which enables them to function as regenerative nano-antioxidants. This redox property allows CeO_2_ NP to directly scavenge superoxide radicals and catalytically decompose H_2_O_2_ in a manner analogous to SOD and catalase, thereby substantially lowering cellular ROS burden and suppressing lipid peroxidation (Celardo et al., 2011). The resulting stabilization of membrane integrity limits oxidative damage and preserves essential cellular processes. Furthermore, reduced ROS accumulation indirectly restrains MG overproduction and likely enhances the efficiency of the glyoxalase system, promoting MG detoxification and alleviating carbonyl stress (Hasanuzzaman et al., 2018).

Under Cr stress, NO signaling was moderately enhanced in rice plants, as evidenced by increased NO content and NOS activity, along with elevated phenolic and flavonoid levels, reflecting the activation of an intrinsic defense mechanism against metal-induced oxidative stress. NO is a pivotal signaling molecule that is rapidly induced under heavy metal stress and functions in redox regulation, metal detoxification, and activation of downstream defense pathways (Corpas and Barroso, 2013; Fancy et al., 2017). However, the relatively modest enhancement of NO signaling and phenylpropanoid-derived metabolites under Cr stress alone suggests that endogenous defense activation was insufficient to fully counteract Cr toxicity. The supplementation of CeO_2_ NPs markedly amplified NO signaling, leading to substantially higher NO content and NOS activity, which in turn promoted the accumulation of phenolic compounds and flavonoids. Mechanistically, CeO_2_ NPs modulate NO homeostasis by stabilizing cellular redox balance through reversible Ce^3+^/Ce^4+^ redox cycling, which limits excessive ROS accumulation and prevents ROS-mediated NO quenching (Bai et al., 2024). This redox buffering preserves NO bioavailability and enables sustained NO signaling under chromium stress (Brazdil, 2022). Elevated NO levels subsequently activate phenylalanine ammonia-lyase and other key enzymes of the phenylpropanoid pathway, promoting the accumulation of phenols and flavonoids that function as chromium chelators, ROS scavengers, and membrane-stabilizing compounds (Twaij et al., 2025). In parallel, NO-dependent post-translational modifications, particularly S-nitrosylation, may fine-tune the activity of antioxidant and secondary metabolic enzymes, further enhancing stress-responsive metabolic efficiency. Collectively, CeO_2_ NP-induced reinforcement of NO signaling operates as a central regulatory node integrating redox homeostasis with secondary metabolism, thereby strengthening rice defense responses and improving tolerance to Cr-toxicity.

Chromium stress imposes severe oxidative and carbonyl stress in rice plants by accelerating ROS generation and MG accumulation, necessitating the activation of an efficient antioxidant and detoxification network. In the present study, CeO_2_ NPs markedly enhanced the activities of antioxidant enzymes, redox metabolites, and glyoxalase system components, with the most pronounced stimulation observed under combined Cr stress + CeO_2_ NP treatment, indicating a synergistic protective response. The progressive increase in SOD, APX, and GPX activities reflects strengthened primary ROS detoxification, enabling rapid dismutation of superoxide radicals and efficient removal of H_2_O_2_ generated under Cr stress (Gill and Tuteja, 2010). The substantial upregulation of MDHAR, DHAR, and GR, together with elevated AsA and GSH levels, highlights the effective reinforcement of the AsA–GSH cycle, which is central to maintaining cellular redox homeostasis under heavy metal stress (Foyer and Noctor, 2011). The enhanced activity of GST further suggests improved detoxification capacity through conjugation of toxic metabolites and lipid peroxidation products. Mechanistically, the redox-active Ce^3+^/Ce^4+^ cycling of CeO_2_ NPs likely lowers excess ROS directly while simultaneously acting as a redox modulator that triggers antioxidant gene expression and enzyme activation, thereby amplifying endogenous defense pathways. In parallel, the significant stimulation of glyoxalase I and II activities indicates efficient detoxification of MG, linking antioxidant defense with carbonyl stress mitigation and preventing MG-induced cellular damage (Hasanuzzaman et al., 2017). Overall, the coordinated enhancement of enzymatic antioxidants, non-enzymatic redox metabolites, and the glyoxalase system under CeO_2_ NP application demonstrates a robust, integrated defense mechanism that restores redox balance, limits oxidative injury, and improves rice tolerance to chromium toxicity.

The accumulation of root structural and metabolic carbon fractions was markedly influenced by Cr stress and CeO_2_ NP application, indicating substantial remodeling of root cell wall architecture and carbon metabolism in rice plants. Chromium stress alone significantly enhanced root cellulose and lignin contents, reflecting stress-induced reinforcement of cell wall components that act as physical barriers limiting metal entry and translocation, while the pronounced increase in soluble sugar content suggests osmotic adjustment and reallocation of carbon resources to sustain root metabolism under toxicity conditions (Panda and Choudhury, 2005). The application of CeO_2_ NP further amplified these responses, both under non-stress and Cr-stress conditions, resulting in the highest accumulation of cellulose, lignin, and soluble sugars when CeO_2_ NP were combined with Cr stress. Mechanistically, CeO_2_ NP–mediated alleviation of oxidative stress likely preserves carbohydrate biosynthesis and promotes carbon partitioning toward cell wall polysaccharides and phenylpropanoid-derived lignin, thereby strengthening root structural integrity and restricting Cr penetration into inner tissues. Enhanced lignification may also facilitate metal immobilization within the apoplast, reducing cytosolic Cr toxicity. Moreover, elevated soluble sugar levels under CeO_2_ NP treatment indicate improved photosynthetic availability and osmoprotective capacity, as sugars function as signaling molecules, ROS scavengers, and energy sources supporting stress adaptation. Collectively, the application of CeO_2_ NPs triggers a coordinated accumulation of cellulose, lignin, and soluble sugars in roots, representing a multi-layered tolerance mechanism. Enhanced cellulose and lignin deposition strengthens the cell wall and promotes apoplastic Cr-binding and immobilization, thereby restricting metal translocation to sensitive tissues. Concurrently, elevated soluble sugar levels support osmotic adjustment, provide metabolic energy, and act as signaling molecules to sustain cellular homeostasis under Cr stress. Together, these interconnected processes enhance root structural integrity, metabolic flexibility, and overall Cr-tolerance in rice plants.

Chromium stress severely impaired ionic homeostasis in rice plants, as evidenced by marked reductions in Na^+^ and K^+^ concentrations in both roots and shoots and a concomitant decline in the K^+^/Na^+^ ratio, reflecting disruption of membrane integrity and ion transport processes under metal toxicity. Chromium is known to interfere with plasma membrane H^+^-ATPase activity and ion channel function, leading to impaired uptake and translocation of essential cations, particularly K^+^, which is crucial for enzyme activation, osmotic regulation, and stomatal function (Gill et al., 2015). The pronounced reduction in the K^+^/Na^+^ ratio under Cr stress observed in this study indicates weakened selective ion transport and loss of cellular ionic selectivity, a hallmark of HM–induced stress. In contrast, the application of CeO_2_ NPs under Cr stress significantly restored ion homeostasis by enhancing Na^+^ and K^+^ accumulation in both roots and shoots and markedly improving the K^+^/Na^+^ ratio relative to Cr-stressed plants. Mechanistically, CeO_2_ NPs likely preserve membrane stability by mitigating oxidative damage, thereby maintaining the functionality of ion transporters and channels involved in K^+^ uptake and long-distance transport. Additionally, improved K^+^ retention may be linked to CeO_2_ NPs–mediated regulation of H^+^-ATPase activity and reduced membrane lipid peroxidation, which together enhance selective ion permeability and ionic balance (Rico et al., 2013; Wu et al., 2017). Restoration of the K^+^/Na^+^ ratio under CeO_2_ NPs treatment is particularly significant, as a high K^+^/Na^+^ ratio is essential for sustaining metabolic activity and stress tolerance. Overall, these findings demonstrate that CeO_2_ NPs effectively alleviate Cr-induced ionic imbalance by safeguarding membrane integrity and ion transport systems, thereby contributing to improved physiological performance and chromium tolerance in rice plants.

**Figure 10:**
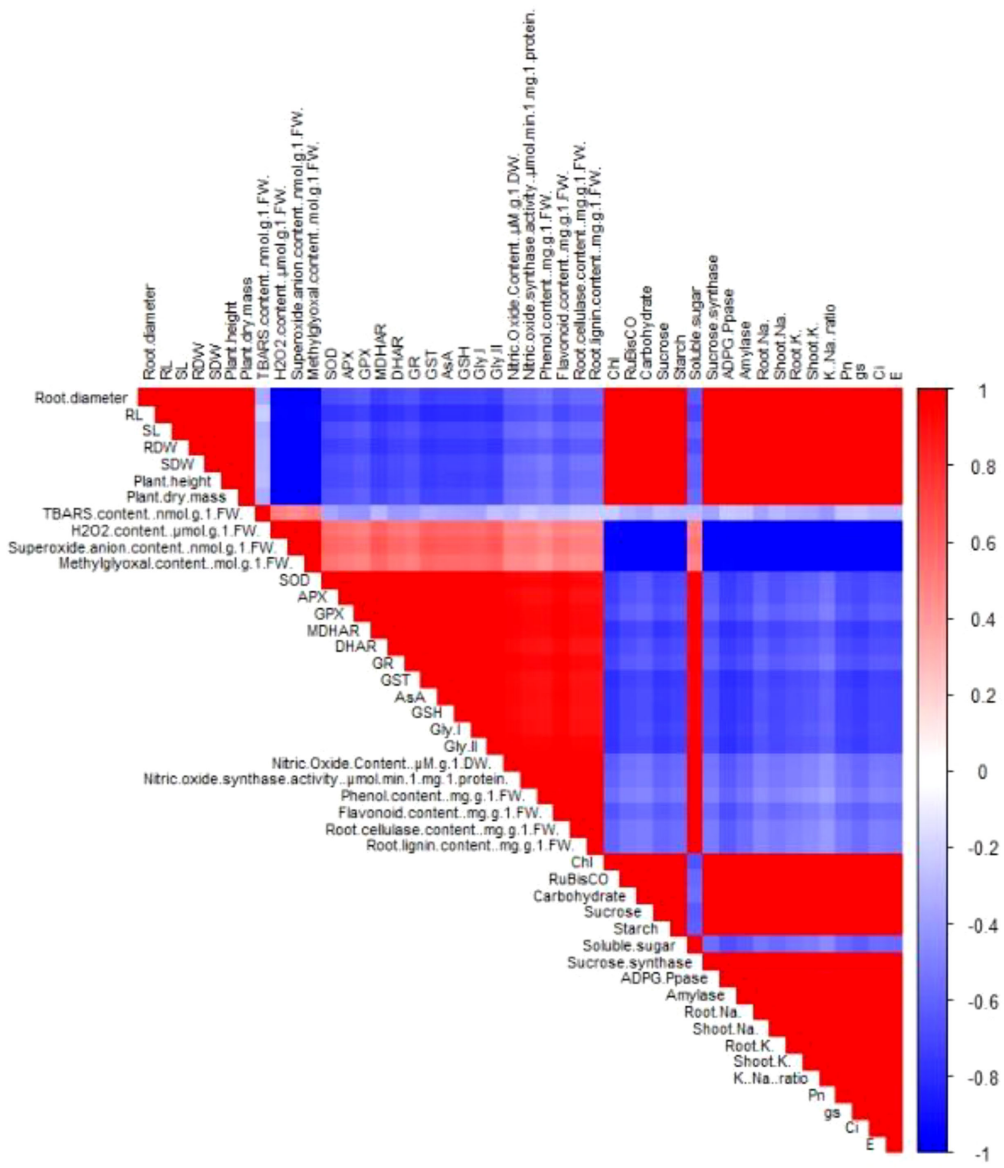
Pearson’s correlation heatmap showing the relationships among morphological traits, oxidative stress markers, antioxidant and glyoxalase system components, signaling molecules, photosynthetic attributes, carbohydrate metabolism, and ion homeostasis parameters in plants.

The correlation analysis reveals a tightly coordinated response among growth, metabolism, antioxidant defense, and ion homeostasis under stress conditions (**Figure 10**). Growth attributes were positively associated with photosynthetic efficiency, carbohydrate accumulation, K^+^ content, and the K^+^/Na^+^ ratio, indicating that sustained carbon assimilation and ionic balance are essential for biomass production. In contrast, growth showed negative correlations with oxidative stress markers, including H_2_O_2_, superoxide, TBARS, and methylglyoxal, highlighting the inhibitory effects of ROS and lipid peroxidation on plant performance. Antioxidant enzymes, non-enzymatic antioxidants, and glyoxalase system components were strongly interrelated and negatively associated with oxidative damage indicators, emphasizing their central role in maintaining redox homeostasis. Photosynthetic traits and carbohydrate metabolism were positively linked with antioxidant capacity, suggesting that efficient ROS detoxification protects photosynthetic machinery and metabolic functions. Additionally, Na^+^ accumulation was negatively correlated with growth and metabolism, whereas K^+^ uptake favored stress tolerance. Overall, the results demonstrate that improved stress resilience arises from the integration of antioxidant defense, metabolic stability, and ion homeostasis.

**Figure 11:**
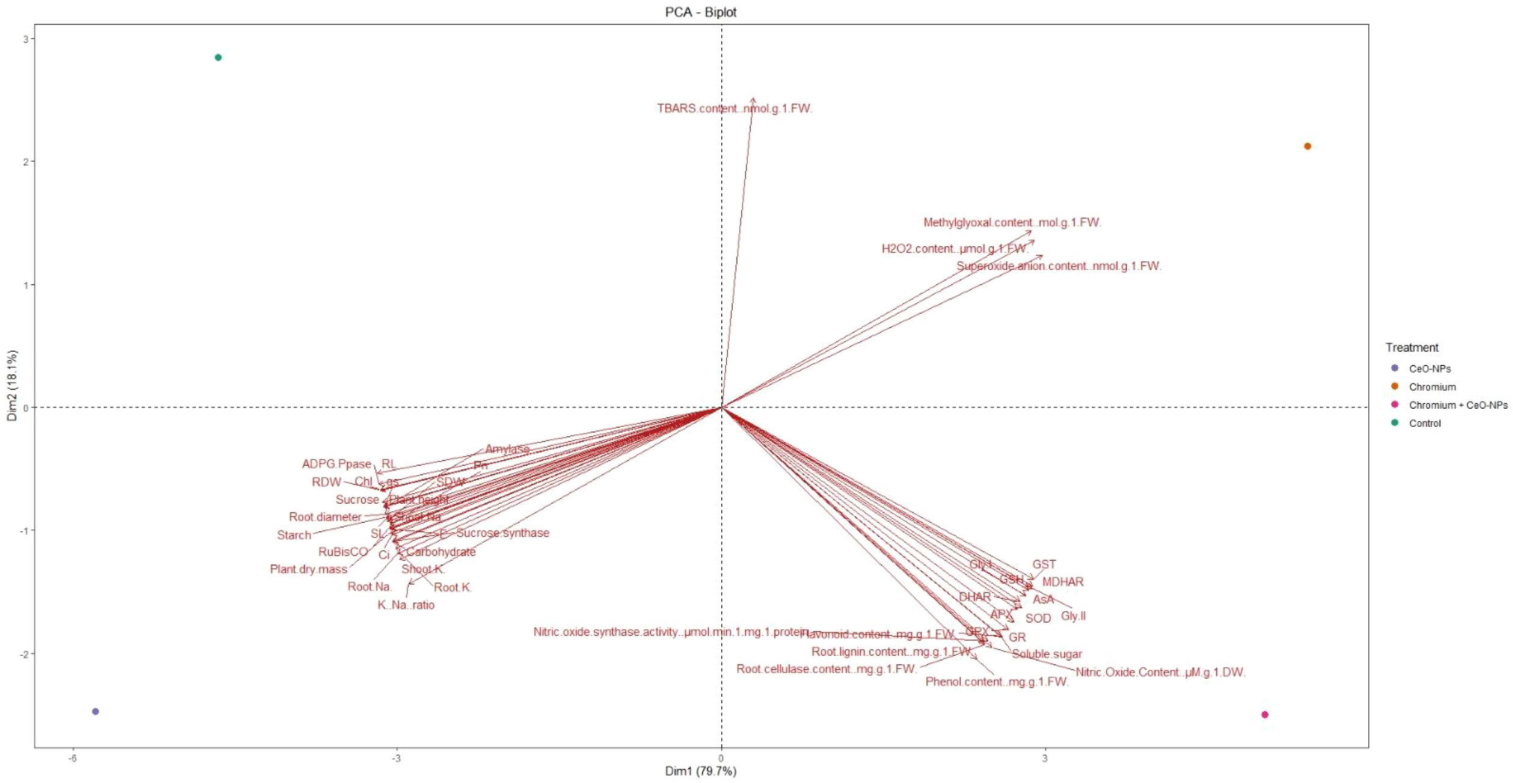
Principal component analysis (PCA) biplot illustrating the multivariate relationships among growth traits, photosynthetic parameters, carbohydrate metabolism, oxidative stress indicators, antioxidant and glyoxalase system components, signaling molecules, and ion homeostasis under different treatments (control, Cr, CeO_2_ NPs, and Cr + CeO_2_ NPs). The first two principal components (PC1: 79.7% and PC2: 18.1%) explain the majority of total variance.

The PCA biplot demonstrates a clear separation of treatments and variables, highlighting the major physiological processes governing plant responses to chromium stress and CeO_2_ NPs application (**Figure 11**). The first principal component, explaining most of the variance, distinctly separates oxidative stress indicators (H_2_O_2_, superoxide anion, TBARS, and methylglyoxal) from growth, photosynthetic, carbohydrate, and ion homeostasis traits, indicating their opposing roles in plant performance. Chromium-treated plants cluster with oxidative damage markers, reflecting severe redox imbalance and growth inhibition. In contrast, CeO_2_ NPs alone and in combination with Cr align closely with antioxidant enzymes, glyoxalase components, signaling metabolites, and metabolic traits, suggesting effective ROS detoxification and restoration of metabolic stability. The association of these treatments with improved K^+^ uptake and K^+^/Na^+^ ratio further emphasizes the role of ion regulation in stress tolerance.

**Figure 12:**
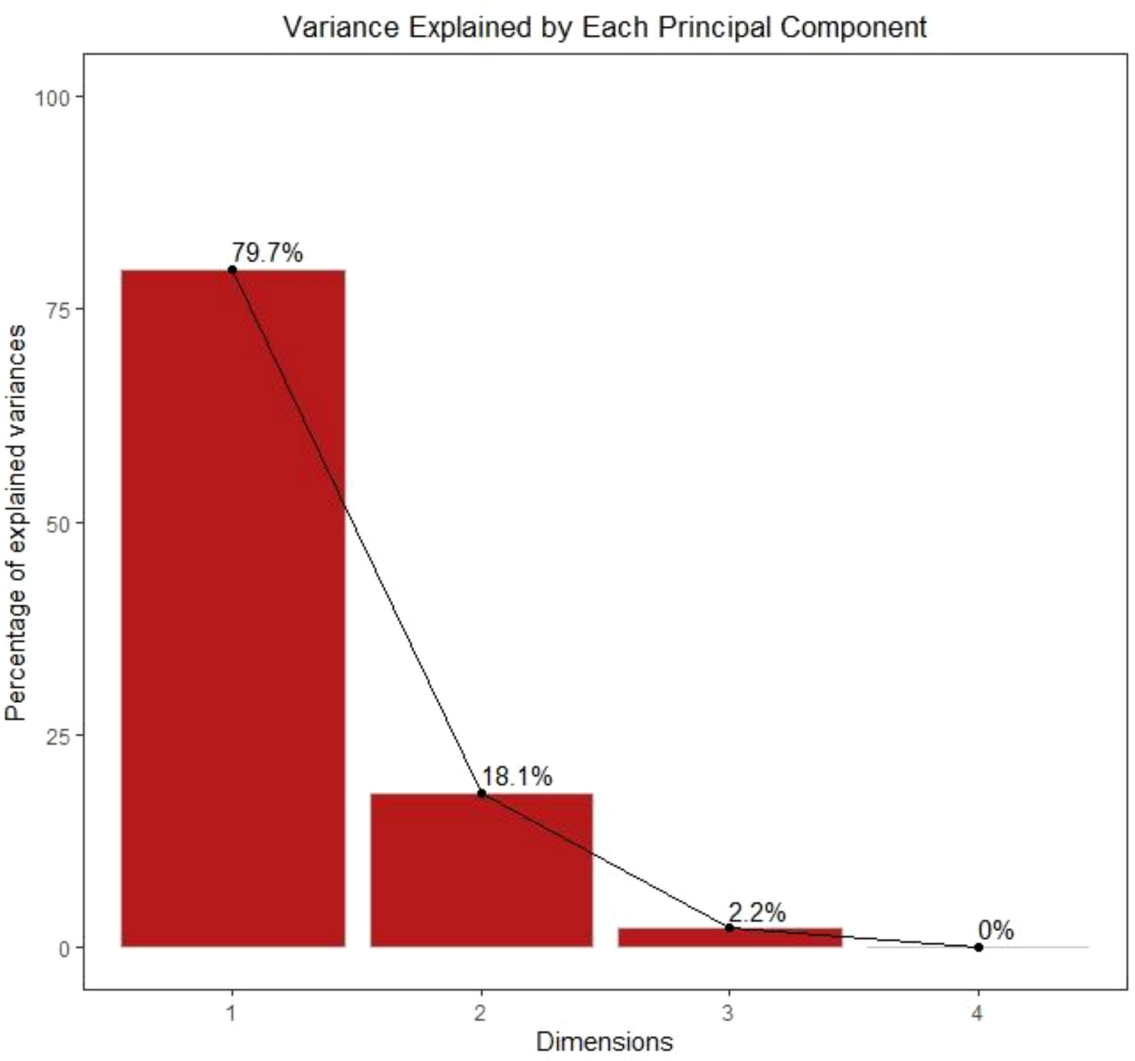
Scree plot showing the percentage of variance explained by each principal component in the PCA. The first principal component (PC1) accounts for 79.7% of the total variance, followed by the second principal component (PC2) explaining 18.1%, while PC3 contributes only 2.2% and PC4 has a negligible contribution. Together, PC1 and PC2 explain 97.8% of the total variability, indicating that the multivariate dataset is well represented by the first two components.

The scree plot clearly indicates that the first two principal components capture almost all of the variability in the dataset, with PC1 and PC2 together explaining 97.8% of the total variance (**Figure 12**). The dominance of PC1 suggests that a single underlying gradient primarily associated with stress intensity and oxidative damage versus growth and metabolic performance drives most of the variation among treatments. PC2 provides additional but smaller discriminatory power, likely reflecting secondary adjustments related to antioxidant regulation and metabolic fine-tuning. The negligible contribution of subsequent components indicates limited additional information beyond PC2, confirming that the complex physiological, biochemical, and ionic responses of plants to chromium stress and CeO_2_ NPs application can be effectively interpreted using the first two principal components alone.

## 5 Conclusion

This study highlights the beneficial role of CeO_2_ NPs in mitigating the adverse effects of Cr stress in rice plants. The results provide valuable insights into the positive influence of CeO_2_ NPs on multiple aspects of plant physiology and metabolism. Chromium stress markedly impaired plant growth, photosynthetic performance, ionic homeostasis, and carbon metabolism. However, the application of CeO_2_ NPs significantly reduced Cr accumulation and its translocation within plant tissues, primarily through enhanced antioxidant enzyme activities and the restoration of key metabolic processes. The observed increase in antioxidant enzyme activities, coupled with a substantial reduction in ROS levels, clearly indicates the ability of CeO_2_ NPs to alleviate Cr-induced oxidative stress. Furthermore, improved NO signaling, enhanced accumulation of secondary metabolites and proteins, and the maintenance of K^+^/Na^+^ homeostasis collectively contributed to improved stress tolerance in rice plants. Overall, the findings suggest that CeO_2_ NPs represent a promising strategy for minimizing Cr toxicity in rice. Future studies should focus on the practical application of CeO_2_ NPs, particularly their potential use as nano-fertilizers under field conditions to mitigate heavy metal contamination in soils and reduce associated environmental and agricultural risks.

## Data Availability

The original contributions presented in the study are included in the article/supplementary material. Further inquiries can be directed to the corresponding author/s.
